# Metastasis to the thyroid gland as the first clinical manifestation of lung cancer – case report

**DOI:** 10.1186/1756-6614-6-S2-A31

**Published:** 2013-04-05

**Authors:** Monika Koziołek, A Sieradzka, E Wentland-Kotwicka, M Machaj, A Makszewska, A Dubińska-Walczak, Anhelli Syrenicz

**Affiliations:** 1 Department of Endocrinology, Metabolic Diseases and Internal Diseases, Pomeranian Medical University in Szczecin, Szczecin, Poland; 2 Clinical Specialized Ambulatory of Endocrinology, Autonomous Public Clinical Hospital No. 1 of Pomeranian Medical University in Szczecin, Szczecin, Poland; 3 Department of Endocrinology and Internal Diseases, Autonomous Public Regional Joint Hospital in Szczecin, Szczecin, Poland; 4 Endocrinology Outpatient Clinic Regional Hospital in Kołobrzeg, Poland; 5 "Medical Care" Non-public Healthcare Facility, Szczecin, Poland

## Introduction

The incidence of metastatic thyroid tumours range from 2 to 3% of all thyroid cancer cases. Histological and immunohistochemical examination plays a decisive role in metastasis recognition. Metastases to the thyroid gland are rarely identified in cytological diagnostics. Patients who were postoperatively recognized with metastasis to the thyroid gland were diagnosed in cytological results as follicular tumour, cancer or atypical cells. We present a case of a 64-year-old male, who was diagnosed with lung adenocarcinoma based on diagnostics of a nodular change in the thyroid gland.

## Case report

A 64-year-old man was admitted to our Outpatient Clinic in 2009 with a chief complaint of a palpable nodule on the right side of the neck. Thyroid ultrasonography revealed a 40 mm solid change in the right lobe of the thyroid gland, therefore an ultrasound-guided fine-needle aspiration biopsy (FNAB) of the thyroid nodule followed by a cytological examination was performed. Because there was a suspicion of a follicular tumour a right-side thyroidectomy was done. Histopathological and immunohistochemical examinations suggested a metastasis to the thyroid gland, most probably from a kidney. An abdominal CT scan which also included bases of the lungs revealed cortical cysts in both kidneys and was showing progression of subpleural tumour in the right lung in comparison to the chest CT scan obtained in 2009. The patient was directed to the Department of Lung Diseases where he was diagnosed as having adenocarcinoma typus bronchioalveolaris. The patient was treated with chemotherapy. In 2011 metastases to the bones were discovered. In January 2012 ultrasonography revealed: three solid lesions in the left lobe of the thyroid gland and a 15 x 10 mm solid hypoechogenic lesion on the right side of the neck. FNAB of the largest nodule in the thyroid gland disclosed benign cells while FNAB of the lesion on the right side of the neck - cellulae carcinomatosae. Since March 2012 there was no more further information about the patient.

## Conclusions

1. a metastasis to the thyroid gland may be the first clinical manifestation of another organ cancer;

2. a suspicion of a follicular tumour in cytological material does not exclude recognition of a metastasis of another organ cancer to the thyroid gland in postoperative histopathological examination.

